# Temporal lobe epilepsy with amygdala enlargement: a subtype of temporal lobe epilepsy

**DOI:** 10.1186/s12883-014-0194-z

**Published:** 2014-10-02

**Authors:** Rui-Juan Lv, Zhen-Rong Sun, Tao Cui, Hong-Zhi Guan, Hai-Tao Ren, Xiao-Qiu Shao

**Affiliations:** Department of Neurology, Beijing Tiantan Hospital affiliated to Capital Medical University, 6 Tiantan Xi Li, Dongcheng District, 100050 Beijing, PR. China; Department of Neurosurgery, Beijing Tiantan Hospital affiliated to Capital Medical University, 100050 Beijing, PR. China; Department of Neurology, Peking Union Medical College Hospital, Peking Union Medical College, Chinese Academy of Medical Sciences, Beijing, P. R China

**Keywords:** Amygdala enlargement, Amygdala volume, Temporal lobe epilepsy

## Abstract

**Background:**

Some recent studies suggest that some imaging-negative temporal lobe epilepsy (TLE) had significant amygdala enlargement (AE). Contradictory data were also reported in previous studies regarding the association between AE and TLE. The present study was to investigate the clinical characters of a group of TLE with AE and compare the amygdala volume of the same patient before and after antiepileptic drugs treatment by a larger sample size.

**Methods:**

This study recruited 33 mesial TLE patients with AE and 35 healthy volunteers. The clinical history, seizure semiology, electroencephalogram (EEG), fluorodeoxyglucose-positron emission tomography (FDG-PET) and amygdala volume were investigated. The amygdala volume were compared between ipsilateral and contralateral sides, TLE patients and 35 healthy controls, and patients at first and follow-up visit by 3.0 T MRI.

**Results:**

Average seizure onset age was 42.0 years (SD 14.3). All patients had complex partial seizures, fourteen had occasional generalized tonic-clonic seizures which often happened during sleep. Ninety percent patients suffered from anxiety or depression. Thirty percent patients had memory decline. Interictal epileptiform discharges appeared predominantly in the anterior or inferior temporal area ipsilateral to AE. Interictal FDG-PET showed regional glucose hypometabolism in the ipsilateral temporal lobe. No hippocampal sclerosis (HS) was suspected in all patients. 22 patients demonstrated good seizure control and significantly reduced volume of the enlarged amygdala after treatment (P < 0.01). The other 11 patients showed initial response to treatment, followed by a gradual increase in seizure frequency over time, and no volume change of the enlarged amygdala after treatment.

**Conclusions:**

TLE with AE probably represents a distinct nosological and probably less homogeneous syndrome which is most likely a subtype of TLE without ipsilateral HS. The chronic and long lasting inflammatory processes or focal cortical dysplasia could lead to amygdala enlargement possibly.

## Background

The amygdala are located in the medial temporal lobe of human brain. They receive extensive nerve input from many different brain areas, such as many sensory areas, hippocampus, hypothalamus, thalamus, frontal lobe and so on [1]. Therefore, they can influence neuroendocrine, emotional, and cognitive aspects of biologic information processing. The amygdala play a crucial role in mediating affective behavior in humans and primates [2]. Some functional imaging studies showed that emotional information processing, for instance angry or happy facial expressions, activated the amygdaloid complex [3,4]. In patients with major depression, there is increased metabolism in limbic areas, indicating an overactivation of the amygdala might be a trait marker of depression [5]. These findings demonstrate that the amygdala are critical brain structures for the emotional evaluation of specific sensory input on the background of individual experience.

Emotional disturbances and psychiatric problems are frequently encountered in patients with temporal lobe epilepsy (TLE) [6]. Previous studies demonstrated significant amygdala enlargement (AE) was associated with dysphoric disorder and psychosis in TLE patients without hippocampal sclerosis (HS) [7,8]. Furthermore, one study proposed that psychosis in patients may develop on the background of the dysphoric disorder of TLE [8]. A neurophysiological study using intracranial recordings showed that 5% of mesial TLE (MTLE) patients had seizure onset in the amygdala [9]. Previous imaging studies reported that isolated unilateral amygdala damage was observed in about 8% of MTLE patients [10]. Therefore, the amygdala may not only be involved and affected in MTLE but may also be regarded as an epileptogenic focus of MTLE.

Recently, some studies reported AE was observed in “magnetic resonance imaging (MRI) negative” TLE [7,8,11,12]. “MRI negative” TLE was defined by the absence of a neocortical lesion, normal hippocampal volumetry, and absence of any increased signal in the mesial temporal lobes on routine visual assessment, despite we cannot preclude completely that “MRI negative” TLE may have HS identified after epilepsy surgery [13]. However, some earlier studies found inconsistent results. For instance, the study performed by Kalviainen R et al. showed that the amygdala were decreased in volume by at least 20% in 19% of TLE patients [14]. Moreover, isolated amygdala sclerosis has been described in some more earlier studies [15,16]. Up to date, a role for the amygdala as a focus of epilepsy has not been explicitly established, unlike TLE with HS. Considering that the above findings are inconsistent and the prior studies are confined to small number of TLE patients, this study aims to investigate the clinical significance of AE in “MRI negative” TLE patients detected by MRI and compare the amygdala volume of the same patient before and after treatment by a larger sample size.

## Methods

### Subjects

We recruited 33 MTLE patients with AE (AE group) in our Comprehensive Epilepsy Center from June 2010 to April 2012. As a comparison for amygdala volume, 35 healthy volunteers (normal group, mean 43.1 (SD 12.3) years) were also recruited from the subjects who underwent 3.0 T MRI study during the same period. Informed consent to participate the study and for publication for clinical details were obtained from each subject enrolled, and the study was reviewed and approved by the ethics committee of Beijing Tiantan Hospital affiliated to Capital Medical University in the People’s Republic of China.

MTLE with AE was diagnosed according to semiology and scalp electroencephalogram (EEG) recording, and by 3.0 T MRI, as described below. 3.0 T MRI confirmed AE in all patients, who had lateralized seizures thought clinically and electrically consistent with an origin in the temporal lobe ipsilateral to AE, as determined by the assessment and agreement of two epilepsy specialists. Patients in whom there was a high suspicion of tumorous disease where the enlarged amygdala apparently compressed the adjacent tissue or showed clear intensity changes in T2 weighed or FLAIR images were excluded. Patients with dual pathologies were also excluded from this study. All patients received 24-hour EEG recordings with sphenoidal electrodes using the 10–20 system of scalp electrode placement. Recordings included both waking and sleeping states. Interictal fluorodeoxyglucose positron emission tomography (FDG-PET) was also performed to support the clinical diagnosis. Localization of the epileptogenic zone was determined by converging seizure semiology, EEG, 3.0 T MRI, and FDG-PET. Time from the first seizure to the date of the present study was at least one year. AE group were followed up for half a year to one year and the amygdala volume were determined again at follow-up visit. In addition, the Montreal Cognitive Assessment (MoCA) was performed to test for cognitive impairment, Hamilton Anxiety Scale (HAMD) and Hamilton Anxiety Scale (HAMA) were used to test for depression and anxiety. We also tested for paraneoplastic or autoimmune limbic involvement in twenty-one patients aged equal to or greater than 40 years old by measuring antibodies against Hu, Yo, Ri, Ma2, CV2/CRMP5, NMDA-R, CASPR2, AMPA1-R, AMPA2-R, LGI1, and GABA2-R. In 12 patients, cerebrospinal fluid (CSF) and serum were tested, while serum only was tested in the other 9 patients who refused lumber puncture. Serum and CSF titers for antibodies against Hu, Yo, Ri, CV2/CRMP5, amphiphysin, and Ma2 were measured by immuno-dot-blotting using a commercial test. The antibodies against NMDA-R, CASPR2, AMPA1-R, AMPA2-R, LGI1, and GABA2-R were measured by indirect immunofluorescence.

### MRI assessment and volumetric measurements

For AE group patients and normal volunteers, an MRI study was performed with a 3 T MR scanner during the same period. To preclude seizure induced brain lesions as far as possible, the scan time interval after seizure were seven days at least. The MRI assessment and amygdala volumetric measurements referred to Mitsueda-Ono’s method [12].

### Statistical analysis

Statistical analyses were performed using SPSS (version 13.0). Quantitative variables were expressed as mean ± SD or n (%). *T* test or Mann–Whitney test for independent samples was used for comparison of quantitative variables according to the test of normality. Amygdala volume were statistically analyzed by paired t tests between AE group and normal controls, the normal and abnormal side for each patient and also analyzed between the larger and smaller sides for the normal control group. We also compared the abnormal side amygdala volume in the same patient of AE group before and after treatment. All tests were performed at a level of significance of 5% (*p* < 0.05).

## Results

### Clinical profiles of AE group

The patient group consisted of seventeen women and sixteen men, with an averaged seizure onset age of 42.0 years (SD 14.3). The duration from the first seizure to this MRI study varied from one year to twelve years (a median of 2 years). No patient had a history of febrile convulsions or early epileptogenic insults. All patients had temporal lobe complex partial seizures (CPSs) beginning with motionless staring and behavioral arrest, followed by oral alimentary or manual automatisms. Total CPS duration ranged from one to five minutes. Six patients reported auras; two reported a strange but indescribable feeling, one smelled blood, and three reported a nervous feeling in their heart. Fourteen of 33 patients had generalized tonic-clonic seizures occassionally, which often happened during sleep. The frequency of CPSs was at least once per month in 32 of 33 patients, while the other patient (patient number 1) had seizure once or twice a year. Ninety percent of patients suffered emotional problems such as anxiety or depression. It was noteworthy that the emotional problems appeared before or simultaneously with seizures. Thirty percent of patients reported memory decline. One patient tested positive for the LGI1 antibody in both blood and CSF, who got great improvement in seizure frequency and memory impairment after hormonotherapy.

In all patients, EEG recordings revealed interictal spikes localized primarily to sphenoidal electrodes or in the anterior temporal-inferior frontal region (F7/F8). Unilateral interictal epileptiform discharges (IEDs) were ipsilateral to the enlarged amygdala in 31 patients with confirmed unilateral AE, while the two patients with bilateral AE (patient number 12 and 16) showed bilateral IEDs. Two patients presented habitual seizures during EEG examination. The ictal EEG recordings showed that seizure onset was ipsilateral to the AE and appeared predominantly on the anterior temporal electrodes and sphenoidal electrodes.

By 3.0 T MRI, AE was detected on the right in fifteen patients, on the left in sixteen and bilaterally in two. The enlarged amygdala was iso- to slightly hyper-intense on 3.0 T FLAIR images and was not enhanced by gadolinium. No HS was suspected in any of these patients as assessed by a neuroradiologist and two investigators (R-J Lv and X-Q Shao). For these patients, interictal FDG-PET showed regional glucose hypometabolism in the anterior temporal lobe, especially in the mesial area and temporal pole ipsilateral to the AE side. The clinical and demographic features of the AE patient group were presented in Table 1.

**Table 1:** **Clinical features of the 33 temporal lobe epilepsy patients with amygdala enlargement**

**Patient Number**	**Age at scan (years)**	**Gender**	**Onset age (years)**	**Seizure type**	**EEG**	**Imaging**	**SGTC time**	**CPS frequency**	**Emotioal disorder**	**AEDs**
1	27	F	20	CPS SGTC	L	L	Sleep	1-2/year	Depression, anxiety	CBZ 400 mg/d
2	25	F	23	CPS	R	R		3-5/month	Depression, anxiety	CBZ 400 mg/d
3	28	M	26	CPS	R	R		2/month	Depression, anxiety	OXC 600 mg/d
4	66	M	61	CPS	L	L		2-4/month	Anxiety	LEV 1000 mg/d OXC 600 mg/d
5	36	F	34	SPS CPS SGTC	L	L	Sleep	3/month	Depression, anxiety	CBZ 400 mg/d
6	22	M	20	SGTC	R	R	Sleep	3-6/month	Depression, anxiety	CBZ 600 mg/d
7	77	F	75	CPS	L	L		4/month	Dementia	CBZ 300 mg/d
8	41	F	40	CPS	L	L		3-6/month	Depression	CBZ 400 mg/d
9	60	M	50	CPS SGTC	R	R	Sleep	3-8/month	Depression, anxiety	LEV 1000 mg/d OXC 400 mg/d
10	49	F	48	CPS	L	L		5/month	Depression, anxiety	CBZ 400 mg/d
11	59	M	57	CPS	R	R		1/month	Depression, anxiety	CBZ 600 mg/d
12	52	F	50	CPS SGTC	B	B	Awake	4-5/month	Depression, anxiety	CBZ 400 mg/d
13	51	F	45	CPS	L	L		7/month	Depression, anxiety	LEV 1000 mg/d CBZ 600 mg/d
14	56	M	55	SPS CPS SGTC	L	L	Awake	3-5/month	Dementia, memory decrease	CBZ 600 mg/d
15	44	M	43	CPS	L	L		3-7/month	Depression, anxiety	CBZ 400 mg/d
16	48	F	43	CPS	B	B		8/month	Memory decrease, bipolar disorder	LEV 1000 mg/d CBZ 600 mg/d
17	61	F	57	SPS SGTC	R	R	Sleep	3-7/month	Depression	OXC 1200 mg/d
18	40	M	39	CPS SGTC	L	L	Awake	7/month	Depression, Anxiety, memory decrease	CBZ 600 mg/d
19	56	M	55	CPS SGTC	L	L	Sleep	3/month	Depression, Anxiety, memory decrease	CBZ 600 mg/d
20	31	M	30	CPS SGTC	L	L	Sleep	3-6/month	Depression, anxiety	OXC 900 mg/d
21	69	M	68	CPS SGTC	R	R	Sleep	5/month	Depression, anxiety	CBZ 400 mg/d
22	51	F	49	SPS CPS	R	R		6/month	Anxiety, depression, memory decrease	LEV 1000 mg/d CBZ 600 mg/d
23	36	F	34	SPS CPS	R	R		3-4/month	Anxiety, aggression	LEV 1000 mg/d CBZ 600 mg/d
24	41	F	40	CPS SGTC	R	R	Awake	3-6/month	Anxiety, memory decrease	OXC 900 mg/d
25	60	M	59	CPS	R	R		3-7/month	Depression, anxiety	CBZ 600 mg/d
26	24	F	22	CPS	L	L		3/month	Depression, anxiety	OXC 900 mg/d
27	40	F	37	CPS	R	R		5/month	Depression, anxiety	LEV 1000 mg/d CBZ 600 mg/d
28	35	M	24	CPS SGTC	L	L	Sleep	2-3/month	Depression, anxiety	LEV 1000 mg/d CBZ 800 mg/d
29	35	M	34	CPS	R	R		3-8/month	Depression, anxiety, personality change	CBZ 800 mg/d
30	38	F	36	SPS CPS	R	R		3-6/month	Anxiety, depression, aggression	LEV 1000 mg/d CBZ 600 mg/d
31	51	M	49	CPS	R	R		7/month	Depression, anxiety, memory decrease	LEV 1000 mg/d CBZ 600 mg/d
32	41	M	39	CPS	L	L		3-5/month	Depression, anxiety, memory decrease	CBZ 800 mg/d
33	35	F	23	CPS SGTC	L	L	Sleep	3-7/month	Depression, memory decrease, bipolar disorder	LEV 1000 mg/d CBZ 800 mg/d

Patient age at time of 3.0 T MRI examination. Mean age was 45.0 years (SD 13.9).

Imaging studies consisted of interictal FDG-PET (fluorodeoxyglucose-positron emission tomography).

*F* female, *M* male, *R* right, *L* left, *B* bilateral, *SPS* simple partial seizure, *CPS* complex partial seizure, *SGTC* secondary generalized tonic-clonic seizure, *AEDs* antiepileptic drugs, *CBZ* carbamazepine, *OXC* Oxcarbazepine, *LEV* Levetiracetam, *d* day.

Before visiting our hospital, nine patients were maintained on valproic acid (200-400 mg/day), seven on carbamazepine (100-300 mg/day), ten patients were treated only with so-called “traditional Chinese medicines” with unknown components and six were untreated with medicines. Carbamazepine (400–800 mg/day) or Oxcarbazepine (600–1200 mg/day) was prescribed to replace previous medications after visiting our hospital. All secondary tonic clonic seizures were well controlled thereafter and CPS frequency declined significantly in 22 of 33 patients. Levetiracetam was added to carbamazepine or oxcarbazepine in the 11 patients who did not show a significant decline in CPS frequency. After half a year to one year follow up, 22 patients became seizure free (drug responsive patient group), however the other 11 patients adding levetiracetam (drug non-responsive patient group) showed initial response to treatment, followed by a gradual increase in CPSs seizure frequency over time. Although the seizure of most patients was not frequent, they continued to experience emotional and memory problems. Fifteen patients were diagnosed with clinical anxiety and/or depression according to ICD-10 criteria and were prescribed selective serotonin reuptake inhibitors.

### Results of 3 T MRI amygdala volumetry

Table 2 showed the amygdala volume of all patients in the AE group and normal group determined by 3.0 T MRI. On the side of the enlarged amygdala with the seizure focus as determined by IEDs (ipsilateral), amygdala volume ranged from 1277.2 mm^3^ to 2046.6 mm^3^ (mean 1694.2 (SD 221.2) mm^3^). On the contralateral side, amygdala volume was smaller and ranged from 651.0 mm^3^ to 1494.2 mm^3^ (mean, 1213.7 (SD 232.6) mm^3^). There was a significant difference between the two sides (p < 0.01).

**Table 2:** **Comparison of amygdala volume among the groups**

	**Unaffected side (mm** ^**3**^ **)**	**Affected side (mm** ^**3**^ **)**	**Affected side follow-up (mm** ^**3**^ **)**
AE group	1213.7 ± 232.6	1694.2 ± 221.2	1306.2 ± 172.4
Responsive AE group	1206.8 ± 226.5	1703.6 ± 228.4	1286.2 ± 202.6
Non-responsive AE group	1224.6 ± 237.7	1686.7 ± 215.3	1659.6 ± 168.2
Normal group	Small side	Larger side	
	1202.8 ± 112.3	1298.2 ± 122.5	

Data is expressed as mean ± SD. AE, amygdala enlargement.

Affected side means the AE side and seizure focus, and unaffected side means contralateral side of seizure focus.

Data for normal group and AE group are shown in table. AE group is divided into two subgroups, drug responsive group (22 patients) and drug non-responsive group (11 patients). Smaller versus larger side in normal group and unaffected versus affected side in the AE group are compared respectively. Larger side in normal group is compared with affected side in the AE group. The affected side before versus after treatment in the AE group and two subgroups is also compared. Details are described in the results section.

In normal subjects (n = 35), the smaller side ranged from 760.3 mm^3^ to 1356.2 mm^3^ (mean 1202.8 (SD 112.3) mm^3^), while the larger side ranged from 836.4 mm^3^ to 1434.2 mm^3^ (mean 1298.2 (SD 122.5) mm^3^). There was no significant difference between the two sides (p > 0.05).

Amygdala volume of the affected side in the patient group and larger side in the normal group were compared, and it was significantly larger in AE group (p < 0.01).

Follow-up 3.0 T MRI was performed in all patients half a year to one year after the first MRI. The affected side amygdala volume at follow-up visit ranged from 930.2 mm^3^ to 1550.4 mm^3^ (mean 1306.2 (SD 172.4) mm^3^). Drug responsive patient group showed significantly decreased volume of the enlarged amygdala after treatment (p < 0.01). However, drug non-responsive patient group demonstrated no volume change of the enlarged amygdala after treatment (p > 0.05).

**Figure 1:**
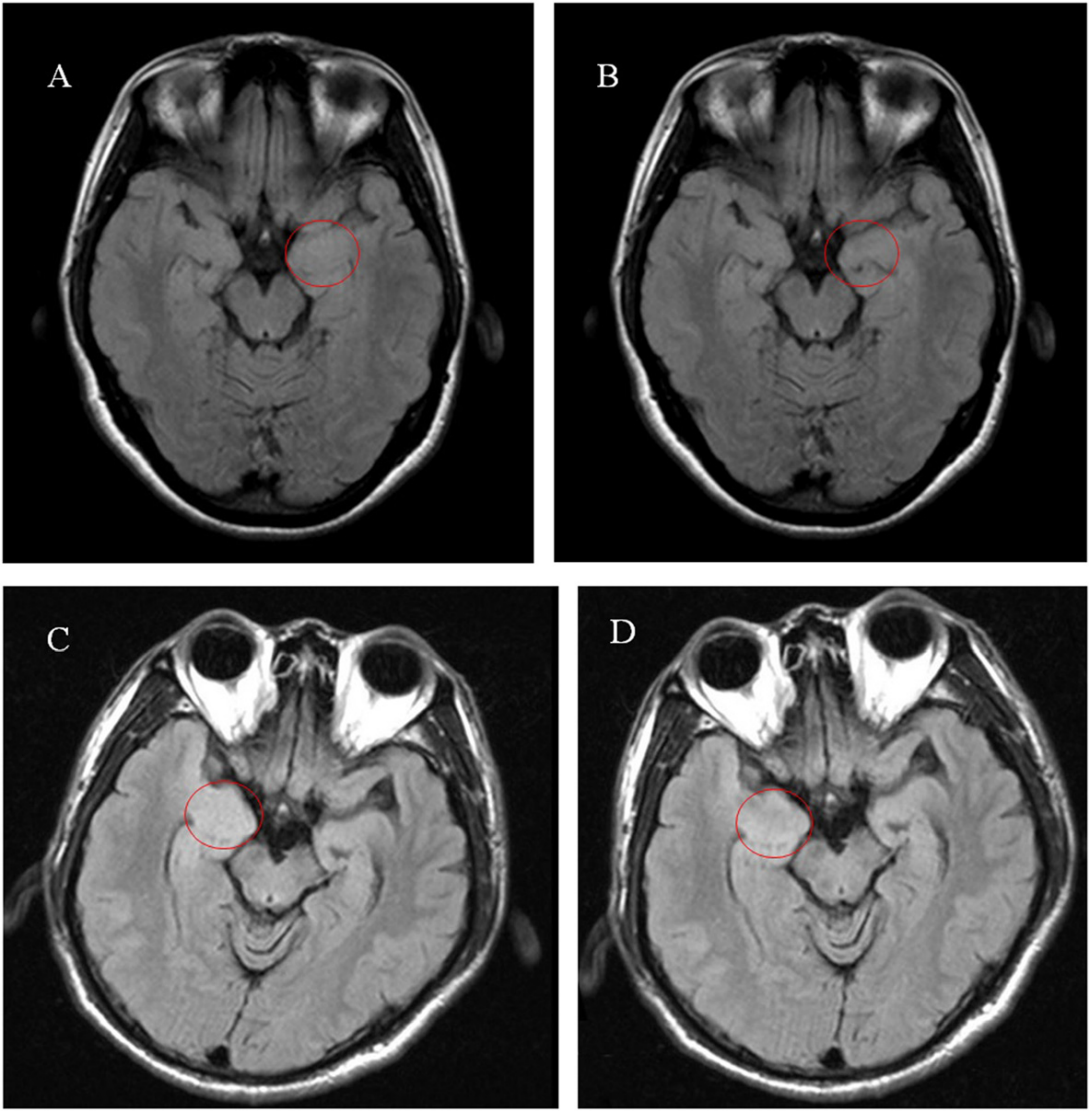
**Axial FLAIR MRI in patient number six (A and B) in drug responsive AE group, and patient number twenty-eight (C and D) in drug non-responsive AE group before and after treatment.** **(A)** Open circle indicates a representative image of left amygdala enlargement in drug responsive AE group. **(B)** Open circle indicates decreased amygdala volume in the same patient after one year of treatment. **(C)** Open circle indicates a representative image of right amygdala enlargement in drug non-responsive AE group. **(D)** Open circle indicates no change of amygdala volume of the same patient after a year treatment.

Figure 1.A showed a representative image of drug responsive AE group by 3 T MRI. Figure 1.B was the image of the same patient after one year treatment (patient number six). Figure 1.C showed a representative image in drug non-responsive AE group. Figure 1.D showed no change of amygdala volume of the same patient after one year treatment (patient number twenty-eight).

## Discussion

Up to date, there have been few amygdala volumetric studies, partly because of the amygdala’s poorly demarcated anatomical boundaries [17,18]. However, the method of amygdala volume measurement used in this study is now well established [7,8,11,12]. The amygdala volumetry was determined in both patients and controls by the same epilepsy specialist (R-J Lv), therefore there was no inter-rater difference or bias. This study is a larger sample size report of TLE patients with increased amygdala volume. We obtained a similar finding in TLE patients with later onset age to the study performed by Mitsueda-Ono T et al. [12]. Our normal ranges and amygdala volume ratios are consistent with recently reported data [12].

In the present study, we found all TLE patients with enlarged amygdala ipsilateral to the IEDs except two patients with bilateral AE showed bilateral IEDs. Although only two patients had ictal EEG, most patients in our study presented unilateral IEDs ipsilateral to AE. Some studies indicated that IEDs in TLE can correlate very well with ictal onset zone, and about ninety percent focal spikes were ipsilateral to epilpeptogenic zone [19,20]. Therefore, we have reason to speculate that most patients of our study presented localized anterior-inferior IEDs always consistent with the side of AE is not accidental phenomena. It may have potential hint that the seizures of 31 patients in our study were lateralized to the hemisphere containing the enlarged amygdala. There was some evidence demonstrating the effect of seizures to MR images which resolved and turned to normal after some time [21,22]. Thus, the decreased volume of the enlarged amygdale at follow-up visit may suggest that AE may be epilpeptogenic zone conversely. The results of this study, combined with previous electrophysiological and radiological studies [11,12,23,24], indicate that AE may function as an epileptogenic focus in this subgroup of TLE patients. However, we did not get direct recording from the amygdala by means of depth electrodes and also we did not have ictal single proton emission computed tomography (SPECT), thus we could not definitively demonstrate that the enlarged amygdala was epilpeptogenic zone. This needs further study according to more direct testimony such as invasive intracranial electrodes recording.

The mechanisms leading to amygdala enlargement in TLE are a matter of debate. A recent study reported the chronic and long lasting inflammatory processes with or without a self-limited course occurred in these TLE patients could lead to amygdala enlargement possibly [12]. Neuroinflammatory processes have been reported to be one of the causes of adult-onset TLE [25]. Autoimmune-mediated encephalitis has been characterized in middle-aged patients with TLE, and some patients demonstrated a self-limited course [25-27]. In our study, one patient had autoimmune limbic involvement and exhibited great improvements in seizure frequency and memory after immunotherapy, which suggested neuroinflammatory processes participated in amygdala enlargement possibly. Whereas, there were not paraneoplastic or autoimmune limbic involvement found in most patients, perhaps because the tests used for paraneoplastic or autoimmune were not comprehensive enough or owing to ethnic difference. That was the cause that we did not give immunotherapy to most patients. The emotional problems appeared before or simultaneously with seizure in this group of patients, which was similar to the report of limbic encephalitis [28], supporting that the etiology was autoimmune inflammation in some patients possibly. In addition, most patients (22 of 33 patients) showed significantly decreased volume of the enlarged amygdala without immune modulating treatment, which was similar to Mitsueda-Ono’s study [12]. Some studies reported that seizure can induce brain lesions [29,30]. Therefore, MRI scan were performed seven days after seizure at least to preclude seizure induced brain lesions as far as possible. Although we cannot preclude seizure related functional change completely because of the short follow-up time, the decreased volume of the enlarged amygdale at follow-up visit may suggest a self-limited course of neuroinflammation. In this study, the seizure onset was late, and FDG-PET scanning was performed within a few years after onset. This may imply that the epileptogenicity in the amygdala was mild and developed slowly. This assumption was consistent with the fact that seizures have been well controlled with a low dose of AEDs instead of immune modulating treatment in these TLE patients with AE. The assumption of long-lasting mild epileptic activity in the amygdale may be a clue to speculate that the possible etiology of AE was the chronic and long lasting inflammatory processes. However, the therapeutic effect declined gradually with follow-up time prolonging in some patients (11 patients), whose amygdala volume did not change after treatment. Therefore, the AE patients in our study could be heterogenous in etiology. Focal cortical dysplasia (FCD) cannot be precluded in some patients completely, especially those patients showing a decline in therapeutic efficacy. Similarly, a recent study found that FCD and low-grade tumors were major causes of late-onset TLE with AE [31]. The speculated reasons are very similar to the the most recent study [32]. However, it must be emphasized that these interpretations are very speculative and until now, there are no generally accepted models that can explain the mechanism leading to the phenomena of enlarged amygdala. This still need be studied further.

TLE with AE may define a subgroup of “imaging-negative” TLE patients that are clinically different to those with HS. Our patients were all late onset and exhibited fewer secondary generalized seizures compared to those patients with HS, consistent with Bruton’s series [33]. With regard to ictal semiology, similar to Mitsueda-Ono’s study [12], complex partial seizures were the most common sizure type. This group of patients had obvious affective disturbances from the onset of the diseases, mainly manifesting as dysthymia, in accordance with the findings of Tebartz van Elst L [7]. The affective disturbance was different from the general meaning of psychosis because they can be well controlled by antidepressant combined with AEDs. After AEDs treatment, most patients became seizure free or showed a dramatic improvement in seizure occurrence. Seizures are currently well controlled on anticonvulsant medication, a dissimilar situation to the usual natural history of TLE with HS, that was the reason we can not get further pathological results to confirm the mechanism leading to AE. In this group of patients, secondarily generalized seizures often occurred at night. To our knowledge, there are no similar findings reported in the literature and we currently have no plausible explanation for this finding. Further research is needed to replicate this finding.

## Conclusion

These findings do have very important implications for the way we think about the diagnosis of “imaging-negative” TLE. TLE with AE probably represents a distinct nosological and probably less homogeneous syndrome which is most likely a subtype of TLE without ipsilateral HS. If there is no apparent HS in TLE patients, AE should be considered first.

## Abbreviations

TLE: Temporal lobe epilepsy
AE: Amygdala enlargement
EEG: Electroencephalogram
FDG-PET: Fluorodeoxyglucose-positron emission tomography
HS: Hippocampal sclerosis
MTLE: Mesial temporal lobe epilepsy
MRI: Magnetic resonance imaging
MoCA: Montreal Cognitive Assessment
HAMD: Hamilton Anxiety Scale
HAMA: Hamilton Anxiety Scale
CPSs: Complex partial seizures
IEDs: Interictal epileptiform discharges
SPECT: Single proton emission computed tomography
FCD: Focal cortical dysplasia
AEDs: Antiepileptic drugs
